# Healthcare personnel exposure in an emergency department during influenza season

**DOI:** 10.1371/journal.pone.0203223

**Published:** 2018-08-31

**Authors:** Ana M. Rule, Otis Apau, Steven H. Ahrenholz, Scott E. Brueck, William G. Lindsley, Marie A. de Perio, John D. Noti, Ronald E. Shaffer, Richard Rothman, Alina Grigorovitch, Bahar Noorbakhsh, Donald H. Beezhold, Patrick L. Yorio, Trish M. Perl, Edward M. Fisher

**Affiliations:** 1 Department of Environmental Health and Engineering, Johns Hopkins Bloomberg School of Public Health, Baltimore, Maryland, United States of America; 2 Division of Surveillance, Hazard Evaluations, and Field Studies (DSHEFS), National Institute for Occupational Safety and Health, Cincinnati, Ohio, United States of America; 3 Health Effects Laboratory Division (HELD, National Institute for Occupational Safety and Health, Morgantown, West Virginia, United States of America; 4 National Personal Protective Technology Lab (NPPTL), National Institute for Occupational Safety and Health, Pittsburgh, Pennsylvania, United States of America; 5 Johns Hopkins Hospital, Adult Emergency Department, Baltimore, Maryland, United States of America; 6 Division of Infectious Diseases, UT Southwestern Medical Center, Dallas, Texas, United States of America; Public Health England, UNITED KINGDOM

## Abstract

**Introduction:**

Healthcare personnel are at high risk for exposure to influenza by direct and indirect contact, droplets and aerosols, and by aerosol generating procedures. Information on air and surface influenza contamination is needed to assist in developing guidance for proper prevention and control strategies. To understand the vulnerabilities of healthcare personnel, we measured influenza in the breathing zone of healthcare personnel, in air and on surfaces within a healthcare setting, and on filtering facepiece respirators worn by healthcare personnel when conducting patient care.

**Methods:**

Thirty participants were recruited from an adult emergency department during the 2015 influenza season. Participants wore personal bioaerosol samplers for six hours of their work shift, submitted used filtering facepiece respirators and medical masks and completed questionnaires to assess frequency and types of interactions with potentially infected patients. Room air samples were collected using bioaerosol samplers, and surface swabs were collected from high-contact surfaces within the adult emergency department. Personal and room bioaerosol samples, surface swabs, and filtering facepiece respirators were analyzed for influenza A by polymerase chain reaction.

**Results:**

Influenza was identified in 42% (53/125) of personal bioaerosol samples, 43% (28/ 96) of room bioaerosol samples, 76% (23/30) of pooled surface samples, and 25% (3/12) of the filtering facepiece respirators analyzed. Influenza copy numbers were greater in personal bioaerosol samples (17 to 631 copies) compared to room bioaerosol samples (16 to 323 copies). Regression analysis suggested that the amount of influenza in personal samples was approximately 2.3 times the amount in room samples (Wald χ2 = 16.21, p<0.001).

**Conclusions:**

Healthcare personnel may encounter increased concentrations of influenza virus when in close proximity to patients. Occupations that require contact with patients are at an increased risk for influenza exposure, which may occur throughout the influenza season. Filtering facepiece respirators may become contaminated with influenza when used during patient care.

## Introduction

Healthcare personnel (HCP) are at high risk for exposure to seasonal and novel strains of influenza during patient care and aerosol generating procedure (AGPs) [1]. A systematic review and meta-analysis of the annual incidence of influenza among healthy adults and HCP found that, compared to adults working in non-healthcare settings, HCP are at significantly higher risk of developing influenza [2]. Another study among 70 HCP in 22 states during the 2009 H1N1 pandemic found that 50% of the subjects were most likely infected within the healthcare facility[3]. These infections likely include patient-to-HCP and HCP-to-HCP transmissions. Among hospital departments, adult and pediatric emergency departments had the highest HCP infection rates [4].

Healthcare resources and HCP are in demand during months when influenza is circulating and such demand is even higher during an influenza epidemic [5]. More HCP are needed when there are more patients to care for, and during outbreaks of contagious disease, some HCP become ill and may actually decrease availability. Therefore, protecting HCP from acquiring influenza is critical to maintain the workforce. Minimizing the risk of transmission of influenza includes vaccinating HCP and utilizing infection prevention precautions such as the appropriate use of personal protective equipment (PPE) [6].

Medical masks are often recommended for HCP who are in close contact with a suspected or laboratory-confirmed seasonal influenza patient [7]. Medical masks are loose-fitting devices and do not offer protection from infectious aerosols. Filtering facepiece respirators (FFRs) are tight-fitting devices recommended for use by HCP when aerosol transmission is a concern, such as when in close contact with patients with novel strain influenza and when performing aerosol generating procedures on patients with seasonal or novel strain influenza [7, 8]. Because FFRs are disposable, single-use devices, supplies can become scarce during periods of heightened need such as an influenza pandemic. Reuse and extended use of FFRs has been recommended as a mechanism to conserve supplies during a pandemic, but risks of FFR reuse have not been fully characterized [8, 9]. Measuring the routes of exposure to influenza within a healthcare facility and contamination of FFRs can assist in the development of PPE recommendations and pandemic protection plans to protect the estimated 18 million HCP in the United States.

To further enhance our understanding of influenza transmission in frontline HCP and to provide data to policy makers, we measured influenza near the breathing zone of HCP, in air and on surfaces within a healthcare setting, and on FFRs worn by HCP working in a healthcare setting. We hypothesized that frequency and concentrations of influenza positive samples would be greater for personal bioaerosol samplers compared to room samplers given the close proximity of participants to patients while administering health care services.

## Methods

### Participant recruitment

A cross-section of 30 HCP with direct patient contact and a variety of job functions (S1 Table) who worked in a busy, inner-city academic adult emergency department (AED) in Baltimore City, were enrolled at the peak (as determined by reports from the Hospital Epidemiology and Infections Control Division) of the 2014–2015 influenza season (29 December 2014 to 9 February 2015). The emergency department was located in a state-of-the-art, new facility with 10 air exchanges per hour on average within patient care areas. The emergency department sees on average 70,000 patients per year with 67 treatment areas. Participants were eligible to be included if they were 18 years of age or older, employed full-time at the AED in a job that required routine close contact with patients presenting with influenza-like illness (ILI) symptoms, and routinely working dayshift hours (7:00 A.M.– 5:00 P.M.). During the 2014–2015 influenza season, the HCP at the AED were to use medical masks during encounters with patients with ILI symptoms as per hospital policy. FFRs were also available and some AED employees were participating in another study which required them to wear FFRs for all encounters with patients exhibiting influenza like illness. For this study, participants had to be willing and able to use their PPE (FFR or medical masks) when in the presence of patients exhibiting ILI symptoms and remain clean-shaven during the six weeks of sampling. The study was approved by both Johns Hopkins University (JHU) and NIOSH Internal Review Boards. Study participants gave informed consent prior to the study. All participants signed consent form prior to enrollment in the study (Internal review board# HSRB 14-NPPTL-03XP).

### Participant requirements

Thirty participants, including nursing supervisors, registered nurses (RNs), certified nursing assistants (CNAs), shift coordinators, clinical technicians, and support staff, were recruited. During each sampling day, five to seven of the 30 participants wore a personal bioaerosol sampler that collected air from their breathing zone (volume within a ten inch radius from the nose/mouth) during six hours of their work shift. Participants were asked to save FFRs and medical masks used during patient encounters. At the end of the six hour sampling period, FFRs and medical masks were collected and returned to a laboratory within the JHU School of Public Health for processing and storage.

### Questionnaire

After wearing the personal air sampler, participants completed a questionnaire (S1 Fig) to assess the frequency and type of interactions with infected patients and coworkers, and describe the attitudes and practices of the participants when attending to patients with ILI. All participants were asked the length of time they were exposed to each patient, whether they used an FFR or medical mask, the occurrence and length of FFR or medical mask use, and whether they performed aerosol-generating procedures (i.e. intubation, respiratory airway suctioning, nebulizing, or nasopharyngeal aspiration). The data from the questionnaires were used to determine, in part, which masks to test based on potential exposures from aerosol generating procedures. The information gained from the questionnaire will be reported in a separate publication.

### Field data collection

Samples were collected three days per week (Monday, Wednesday, and Friday) and consisted of room and personal bioaerosol samples, used FFRs and medical masks, surface samples, and daily questionnaires. We were provided with de-identified information about the location of patients with confirmed influenza (identified by a diagnostic rapid test) as well as the HCP that had contact with them.

#### Air samples

Personal and room bioaerosol samplers consisted of an button sampler [10] (SKC Inc. Eighty Four, PA) that contained a 25 mm filter (Fluorophore^™^ PTFE, 3.0 μm pore size, Millipore Sigma, Darmstadt, Germany) connected to a personal sampling pump (XR5000, SKC Inc, Eighty Four, PA). The personal sampling pump was wrapped in 2.54 cm thick noise-dampening material (5692T13, McMaster Carr, Aurora, OH) and housed in a personal backpack (CamelBak Products, LLC, Petaluma, CA) with the inhalable sampler clipped in the front strap of the backpack, in the subject’s breathing zone. Room sampling pumps were placed in a modified case (Storm iM2200, Pelican LLC, Torrance, CA) and connected via a 0.9 m Tygon^™^ tube (6.35 mm I.D.) to the inhalable sampler, which was taped to a wall approximately 1.5 m above the floor. Before each sampling period, each of the pumps was calibrated in the lab to 4±0.2 liters of air per minute (L/min) with an electronic flow calibrator (Bios DryCal, SKC Inc., Eighty Four, PA); flow rates were checked and recorded at the end of the sampling period.

Assignment of personal bioaerosol samplers was based on participant availability as determined by a shift schedule provided by management of the adult ED. We attempted to equalize the number of shifts monitored for each participant over the study period. Room air sampler locations were identified with hospital staff in advance to prevent interfering with daily activities. Room samplers were located in each of the two waiting areas, two of the four screening rooms, one of two triage areas and the ED observation unit (emergency acute care unit or EACU) for the same period of six hours as participants wore the personal air samplers. Immediately after the six hours of sampling, all samples were transported to a laboratory within the Johns Hopkins University School of Public Health, where each filter recovered from the sampler was processed as follows: transferred into a 15 ml tube containing 1 ml modified Hank’s Balanced Salt Solution (mHBSS) (Invitrogen, Carlsbad, CA) containing 0.1% bovine serum albumin (BSA) (Sigma-Aldrich, St. Louis, MO), vortexed for 60 seconds, and stored at -20°C until shipment to a laboratory within NIOSH’s Health Effects Laboratory Division (HELD) for analysis.

Temperature and relative humidity data loggers (HOBO U10-003, Onset Corp, Bourne, MA) were deployed with each of the six room samplers. Data loggers recorded every minute.

#### Surface samples

Common high-contact, non-porous hard surfaces (chair surfaces in the triage area and two screening rooms, and the sink area) in the vicinity of room air samplers were swabbed using moistened sterile swabs (Copan Diagnostics, Corona, California). A 10 cm x 10 cm template was used to delineate a 100 cm^2^ swipe area for large surfaces. For non-flat surfaces (e.g. sink faucet handles), 100 cm^2^ areas were estimated. Each swab was then placed in a 15 ml tube containing 1 ml mHBSS, vortexed for 60 seconds, and stored at -20°C.

#### FFRs and medical masks

Subjects who were fitted with a personal bioaerosol sampler were provided with labeled, zip-seal bags and collection bins to place FFRs and medical masks worn during the sampling interval. All bags were stored at -20°C until shipment to the NIOSH (HELD) laboratory.

#### Quality control

For quality control, one positive and one negative control was prepared for each sample type (personal bioaerosol, room aerosol and surface samples). For positive field controls, bioaerosol sampler filters and surface swabs were inoculated with reference influenza virus H1N1 strain A/WS/33 (catalog number VR-825, American Type Culture Collection, Manassas, VA). Negative field controls for air samples consisted of a filter from a prepared aerosol sampler handled in the same way as all of the other samples except that the sampler did not have any air drawn through it. Surface swipe negative controls were handled in the same way as all other surface samples except no surface was swabbed. Positive and negative field control samples served as indicators of field sample integrity associated with handling and shipment of the samples. All samples were kept at -20°C.

### Analytical methods

All samples (bioaerosol filters and surface samples in mHBSS, and FFRs and medical masks in plastic bags) were overnighted in weekly batches at 4°C to the NIOSH HELD laboratory, where samples were stored at -80°C until analysis. Before analysis, four 25 mm diameter coupons were punched out from the mouth area of each FFR and placed in 8 ml of mHBSS. Virus was eluted from all samples by overnight incubation at 4°C in mHBSS.

After overnight incubation, viral RNA was isolated from all samples using the MagMax^™^-96 Viral RNA Isolation Kit (Applied Biosystems/Ambion, Austin, TX) as previously described [11]. The final viral RNA volume was 32 μl. Viral RNA was immediately transcribed into cDNA using the High Capacity RNA to cDNA Master Mix in accordance with the manufacturer’s instructions (Applied Biosystems, Foster City, CA). The final cDNA volume was 40 μl.

The presence of influenza A was evaluated by quantitative Polymerase Chain Reaction (qPCR) assays (Applied Biosystems 7500 Fast Real-Time PCR System). For qPCR determination of total matrix gene or hemagglutinin (HA) gene copies of viral RNA, matrix-specific primers were used as described before [12]. Samples below the qPCR limit of quantification were further analyzed using gel electrophoresis using NuSieve GTG agarose gel (Lonza Inc., Allendale, NJ) along with 10 μl of a 100 base pairs (bp) DNA ladder (N3231L, New England Biolabs, Ipswich, MA).

### Limit of quantification and limit of detection

The limit of quantitation (LOQ) of the qPCR assay was 15 viral copies per PCR reaction tube, which corresponded to a threshold cycle (Ct) value of 34.5 cycles. The limit of detection (LOD) by qPCR was 10 viral copies per sample (Ct value of 35.8 cycles). In cases where a PCR product was detected but the Ct value was higher than the Ct value for the LOQ, the PCR product was evaluated by electrophoresis in a 4.5% NuSieve GTG agarose gel to verify that the PCR product was the correct size (101 bp) for the M1 matrix gene. The LOD with this additional step was two viral copies per sample. A sample containing 2–14 PCR copies of the matrix gene was considered positive for influenza if the product was confirmed to be the correct size by gel electrophoresis. For additional verification, DNA sequence analysis was performed on randomly-chosen samples by a commercial laboratory (Genewiz LLC, South Plainfield, NJ) using predefined Sanger DNA sequencing.

### Calculations

Airborne virus concentrations (*C*_*a*_) were calculated from copies of influenza virus measured from bioaerosol sampler filters (*V*_*f*_) using Eq 1

$$C_{a}=(\frac{V_{f}}{T*Q})*1,000$$ (1)

where *C*_*a*_ = aerosol concentration (virus copies/m3); *V*_*f*_ = # of viruses on filter (copies); *T* = Sampling time (minutes); *Q* = pump flow rate (L/min); and 1,000 is a multiplier to convert from liters to cubic meters.

Surface concentrations (*Cs*, *in units of # of copies/cm*^*2*^) were calculated from copies of influenza virus measured from pooled swipe samples (*Vs*) using Eq 2

$$Cs=\frac{\left(Vs\;x\;DF\right)}{Sa}$$ (2)

where *Cs* = surface concentration (virus copies/cm^2^); *Vs* = # of viruses on pooled swab samples (virus copies); *DF* = dilution factor (16); and *Sa* = surface area swiped (cm^2^).

### Statistical analysis

The study was powered to detect an 18% difference between personal and room bioaerosol samplers. These values were estimated from the average min and max detection rates for influenza positive personal and room aerosol samplers reported in the literature [11, 13–19].

Descriptive statistics (median, range, frequency) and inferential statistics were performed in SPSS version 24 (IBM Corp. Armonk, New York). A regression based Generalized Linear Model approach assuming a Negative Binomial distribution was used to estimate the mean difference between personal and room bioaerosol samples given the dependent variable (virus concentration) was log-normally distributed. The Negative Binomial distribution was selected based on the nature of the data and through superior fit statistics when compared to the normal and Poisson distribution counterpart. Within the statistical model the origin of the sample (personal or room) was entered as a categorical variable with room bioaerosol as the reference category.

## Results

Influenza was detected on 42% of the personal bioaerosol samplers collected. The quantity of influenza copy numbers ranged from 17 to 631 copies per sample (Table 1), equivalent to a 6-hour exposure to aerosols with concentrations roughly from 11 viruses/m^3^ to 438 viruses/m^3^. Influenza was detected in 43% of the room bioaerosol samplers. The number of influenza copies recovered in the sampled rooms ranged from 16 to 323 copies (Table 2), equivalent to 6-hour exposure to aerosols with 13 viruses/m^3^ to 224 viruses/m^3^. These descriptive statistics resulted in a significant difference in the mean influenza virus copies between personal (M = 173.13) and room samples (M = 74.79), B = 0.84, Incident Risk Ratio (IRR) = 2.32 (95% OR CI, 1.54–3.48), Wald χ2 = 16.21, p < .001. This IRR suggests that the amount of influenza in personal samples was approximately 2.3 times the amount in room samples.

**Table 1:** Influenza concentrations in individual personal bioaerosol samples over the study period.

Date	Observations (n =)	No. of positive samples	Copies of influenza RNA/filter		Estimated 6 hour exposure concentration (viruses/m^3^)
			Median	Range	
12/29/2014	6	1	128	128	89
12/30/2014	7	2	23	17–29	15
1/2/2015	7	0	-	<LOD	-
1/5/2015	6	1	86	86	60
1/7/2015	8	7	249	202–326	172
1/9/2015	8	7	267	31–362	186
1/12/2015	8	3	29	24–261	13
1/14/2015	8	8	235	151–326	164
1/16/2015	4	0	-	<LOD	-
1/19/2015	6	0	-	<LOD	-
1/21/2015	4	0	-	<LOD	-
1/23/2015	7	0	-	<LOD	-
1/26/2015	6	2	54	50–60	38
1/28/2015	7	3	91	40–93	63
1/30/2015	6	3	49	39–210	35
2/2/2015	7	6	40	17–339	28
2/4/2015	7	6	233	63–381	161
2/6/2015	6	2	339	47–631	236
2/9/2015	7	2	39	17–61	28

**Table 2:** Room bioaerosol sample results by day.

Date	No. of positive samples	No. of rooms monitored*	Copies of influenza RNA/filter	
			Range	Median
12/29/2014	0	4	<LOD	-
12/30/2014	1	4	16	16
1/2/2015	0	4	<LOD	-
1/5/2014	0	4	<LOD	-
1/7/2015	0	1	<LOD	-
1/9/2015	2	2	35–82	59
1/12/2015	2	3	16–31	24
1/14/2015	4	4	20–46	35
1/16/2015	3	3	40–179	83
1/19/2015	2	4	19–24	22
1/21/2015	4	4	37–323	101
1/23/2015	4	4	20–127	39
1/26/2015	3	4	23–27	25
1/28/2015	0	4	<LOD	-
1/30/2015	1	4	43	43
2/2/2015	0	4	<LOD	-
2/4/2015	1	4	159	159
2/9/2015	1	3	44	44

* Included only four rooms that had healthcare worker occupancy. Two waiting areas excluded.

The frequency of positive personal bioaerosol samples was greatest for CNAs and RNs (Table 3). Nursing supervisors had the highest median copy number (440 copies, or 250 viruses/m^3^) on their personal sampler filters, while sampler filters from CNAs had the lowest median copy number with 31 copies or 20 viruses/m^3^. There was a positive correlation (R = 0.83, P = 0.039) between the ratio of the number of self-reported contacts with patients and/or coworkers exhibiting ILI per the number of shifts monitored and the frequency of positive filters for each occupation (Table 3).

**Table 3:** Frequency of positive personal bioaerosol samples by occupation.

Occupation	No. of positive samples	Ratio of patients exhibiting ILI / No. shifts monitored	Total shifts monitored	Frequency of positives	Copies of influenza RNA/filter	
					Median	Range
Support staff	5	1.19	18	28%	212	53 to 269
Shift coordinator	1	1.25	4	25%	39	39
Registered nurse	31	2.33	61	51%	140	17 to 381
Nursing supervisor	2	0.17	6	33%	440	249 to 631
Clinical technician^1^	12	1.10	32	38%	225	17 to 339
Certified nursing assistant	3	3.00	4	75%	31	29 to 61

^1^ For a description of all job titles please refer to S1 Table

The frequency of detection, range, and median number of copies of influenza virus measured on room bioaerosol samples for each patient care area and patient waiting areas sampled (Table 4) ranged from 35% for the observation unit to 56% for screening rooms and 44 to 47% of triage areas. The observation area had the highest level of contamination with 323 copies/filter (224 viruses/m^3^), although waiting area one had the highest median of 81 copies/filter (56 viruses/m^3^). Waiting areas were similar to the other sampled areas, with 41 and 35% frequency of detection, and between 20 and 307 copies of virus detected per filter.

**Table 4:** Frequency of identifying influenza in room aerosol samples in an adult emergency department.

Room	No. of samples collected	No. of positive samples	Frequency of positive samples	Copies of influenza RNA/filter	
				Range	Median
Observation Unit^1^	17	6	35%	26 to 323	69
Screening room C	15	7	47%	19 to 159	23
Screening room D	16	9	56%	20 to 69	40
Triage Area	16	7	44%	16 to 127	33
Waiting Area 1	17	7	41%	24 to 186	81
Waiting Area 2	17	6	35%	20 to 307	42

^**1**^ EACU = Emergency Acute Care Unit

During eight of the 125 shifts sampled, a participant was in contact with a confirmed influenza patient. Of these eight participants (Table 5), four had positive personal bioaerosol samplers with a copy number ranging from 29 to 321. One participant, a RN, had contact with five patients with confirmed influenza. The personal bioaerosol sampler for this participant was positive for influenza with 128 copies (89 copies/m^3^), while a participant with one contact had the highest copy number (321 copies) detected on their personal bioaerosol sampler filter. The frequency of positive personal bioaerosol samplers was not significantly different (p>0.05) between participants that had or did not have contact with influenza confirmed patients.

**Table 5:** Personal bioaerosol sample results of participants that had contact with influenza positive patients.

Date	Occupation	Number of contacts	No. of copies of influenza RNA	Copies of influenza/m^3^
12/29/2014	RN^1^	5	128	89
12/30/2014	CT^2^	1	-	
12/30/2014	RN	3	29	20
1/5/2015	RN	1	86	60
1/5/2015	RN	1	-	
1/5/2015	RN	1	-	
1/9/2015	RN	1	321	223
1/23/2015	CT	1	-	

^1^RN = Registered Nurse;

^2^CT = Clinical Technician

A total of 25 participants performed AGPs while wearing a personal sampler, including an intubation, four airway suctions, 29 nebulizer treatments, and five nasopharyngeal aspirations (Table 6). Of the 25 participants who performed an AGP, influenza was isolated from the personal bioaerosol samplers of 10 participants, with copy numbers ranging from 29 to 321 (20 to 223 viruses/m^3^). None of the bioaerosol samplers worn by the three clinical technicians and all ten of the samplers worn by the RNs who performed AGP had an influenza positive personal bioaerosol sampler.

**Table 6:** Influenza identified in personal bioaerosol samplers after performing aerosol generating procedures.

Occupation	No. of participants	Aerosol generating procedures performed				Influenza identified in personal samples
		Intubations	Airway suctioning	Nebulizer	Nasopharyngeal aspirations	
Clinical Technicians	3	0	1	2	0	0/3 (0%)
Registered nurses	22	1	3	27	5	10/22 (41%)
Total	25	1	4	29	5	10/25 (36%)

A total of 12 FFRs were analyzed for influenza, eight of which were from participants exposed to patients with confirmed influenza. Of these, influenza was identified in three (25%). Four FFRs were tested from a subject who performed an AGP and had a positive personal bioaerosol sampler, yet all four of the FFRs were negative for influenza.

Influenza was identified in 14 of the 30 pooled surface samples (47%) collected from patient care areas (Table 7). Eight of 12 samples from the screening rooms (66%), and four of 12 (30%) samples from triage chairs were positive. Influenza was also detected in two of six (33%) of triage sink samples. The samples ranged from 0.5 to 6.1 virus/cm^2^. The highest concentrations were found in the screening room chairs, with 6.1 and 3.6 virus/cm^2^, in each of the two screening rooms.

**Table 7:** Influenza virus detected in surface samples by area where sample was collected and type of surface swabbed.

Sample area and surface type	# pooled samples	# positive samples	%	Copies of influenza RNA/cm^2^	
				(median)	(range)
Screening room C Chair	6	6	100%	1.9	1.0 to 6.1
Screening room D Chair	6	2	33%	3.5	3.3 to 3.6
Triage back Chair	6	1	17%	0.5	0.5
Triage front Chair	6	3	50%	0.5	0.3 to 1.5
Triage sink	6	2	33%	0.8	0.7 to 0.9
Total	30	14	47%		

## Discussion

Our data demonstrated that the air and surfaces in the hospital adult emergency department are contaminated with influenza virus and that the frequency of virus detection was positively associated with the frequency of contacts with patients or coworkers exhibiting ILI. We identified influenza in the breathing zone of HCP, in room air, and on surfaces within a healthcare setting, and on FFRs worn by our participants. Our data support that exposure to influenza via aerosols (small particles that can be transmitted long range) is likely for HCP working in an emergency room. In addition, the proportion of positive surface samples suggest that contact exposure is also likely.

The 2014/15 influenza season in the U.S. was moderately severe with influenza A H3N2 circulating as the predominant strain along with H1N1 and influenza B [20, 21]. Influenza vaccination for the 2014/15 flu season offered reduced protection against the influenza A H3N2, and had the lowest estimated vaccine effectiveness (19%) since the 2004/05 influenza season [22]. Hospitalization rates for influenza were higher during the 2014/15 influenza season compared to previous seasons [21] and medically attended cases were higher than the previous four influenza seasons [23]. The peak of the flu season occurred in late December (the week ending 27 December 2014), which coincided with our initiation of field sampling [20].

Our study is one of the first to detect the presence of influenza in a healthcare setting spanning from the peak (15 patients with confirmed influenza on Dec 29, 2014) to the tail end of the influenza A season (2 patients with confirmed influenza from February 1-February 9). Influenza was detected during 17 of 19 sampling sessions (89%). Other studies have detected influenza using personal and room aerosol samplers, but few report detection of influenza over multiple days spanning from the peak to the end of the influenza season. Similar to our study, Lueng et al. conducted sampling during the 2014/15 influenza season from December 16 through February 9 in a hospital in Hong Kong and detected influenza virus in five of 10 (50%) sampling periods where patients with influenza A were present [16]. However, the dates on which influenza was detected were not reported and cannot be linked to the timing of the peak of the influenza season in Hong Kong. Lindsley et al. reported influenza positives in air samples for 10 out of 11 days in an urgent care facility in February 2009 [24]. Influenza RNA was detected in 19% (four of 21) of personal aerosol samplers and 46 of 264 (17%) of room samplers, reported to be near the peak of the seasonal influenza season which occurred in mid-February [25]. Blachere et al. reported 42% (three of seven) influenza positive personal samplers worn by physicians at the emergency department at the West Virginia University Hospital during February 2008, on or near the peak of the influenza season [11, 26].

We hypothesized that the personal bioaerosol samples would be positive more often, and with greater concentrations of virus, compared to room bioaerosol samples due to the close proximity of the test subjects to influenza patients when administering patient care. The results were similar for the percentage of positive samples for personal (42%) and room bioaerosol samplers (43%); however, the concentration levels for personal bioaerosols were much higher and significantly so, OR = 2.3, p <0.001. In other studies, virus has been detected in higher concentrations near patients’ heads compared to locations that are more distant to the patient [13]. To the contrary, Cummings et al. reported that influenza was detected in higher concentrations outside the patient room compared to within the patient room where AGPs were being conducted [14]. Similarly, Blachere et al. detected higher concentrations of influenza in the waiting area of a healthcare facility (460 to 16,278 copies/filter) compared to personal bioaerosol samplers (309 to 4,623 copies/filter) [11]. Our results may differ from those of Blachere and Cummings due to difference in layouts within the areas sampled, different care procedures, differences in ventilation, and difference in influenza season characteristics as mentioned above.

Regarding frequency of exposure by occupation, we found that CNAs and RNs had the highest exposure, with 75% of CNA personal bioaerosol samplers and 49% of RN bioaerosol samplers testing positive for influenza respectively. Our results demonstrate a strong positive correlation (Pearson Correlation, R = 0.83, P = 0.039) between the average number of patients per shift and the frequency of the number of positive samplers by occupation. CNAs and RNs had the highest ratio of patient contacts per shift monitored and our results confirm that they are at higher risk of exposure. Findings from other studies [27] indicate significantly increased odds for an influenza A (H1N1) infection among HCP with > 5 contacts to suspected infected patients compared to HCP with < 5 contacts (OR = 1.47, 95% CI = 1.11 to1.94).

Of the eight participants that had contact with patients diagnosed with influenza (Table 5), four (50%) had influenza detected in the personal bioaerosol samplers. Ten of 25 (40%) of samples from participants that reported performing AGPs, were collected from RNs. Few studies have looked at exposure risk during AGPs. In one meta-analysis during the SARS epidemic, Tran and et al. (2012) reported that the most consistent association with increased risk of SARS transmission across multiple studies was identified with tracheal intubation [28]. During the six weeks of our study, only one participant reported performing an intubation. The most common AGP reported by our participants was nebulization. This is consistent with several papers that find nebulization to be one of the most frequently performed procedures (along with mechanical ventilation) [29, 30]. (ref). This is clinically and practically important, since more patients use nebulizers and oxygen masks than undergo bronchoscopy, intubation, sputum induction and CPR. Furthermore, a recent study by Li and colleagues (2017) suggests that nebulization may be the AGP that generates the largest number of aerosol particles. Further studies are needed to confirm Li’s findings, characterize particle composition and determine probability of transmission [29].

We detected influenza contamination of FFRs (3 of 12 analyzed; 25%) used by HCP in an outpatient, yet high-risk clinical setting while administering patient care. A previous study at a student health clinic did not find any influenza among the 43 medical masks analyzed that were worn by HCP [31]. Other studies have reported bacterial and fungal contamination of medical masks used by HCP [32] and bacterial contamination by cleaning staff within a healthcare facility [33] The low copy number of influenza present on the FFRs may be due to the short wear time, which coincides with the length of time the HCP was in close contact with a patient exhibiting ILI.

Surface sampling for influenza and other viruses has been inconsistent in the few studies available. Tang et al. [19] were unable to detect influenza virus RNA on surfaces that were contaminated via direct cough by subjects with confirmed influenza. Killingley et al. (2011) collected samples from fomites to assess person-to-person transmission and found that 19% of fomites were PCR positive for influenza virus [34]. An additional study by Killingley et al. (2016) found that 33 of 671 (4.9%) of the surface swabs detected influenza Type A (H1N1 pandemic 09) by PCR [35]. In contrast, our study found an average 60% positive surface samples, although our detection rate was likely improved because samples were pooled over a week. This strategy may better reflect the risk of cross contamination to HCP. Furthermore, negative room samples could be explained by previous and recent environmental cleaning, which is performed frequently at the Hospital, but we do not have available data per room.

Our study has several limitations. We used PCR technology to identify influenza virus and hence could not differentiate whether the virus identified was viable or not—a consideration when policy recommendations are developed. In addition, the threshold of airborne influenza virus that needs to be inhaled to cause infection is not known definitively and likely varies between individuals based on their previous exposure/immunological history [36, 37]. We only analyzed 12 of 128 FFRs (9%) and none of the 205 medical masks collected due to budget and timing limitations. We analyzed FFRs that had the greatest potential to be contaminated, by selecting FFRs worn by participants who had contact with patients with confirmed influenza, had positive personal bioaerosol samplers, and performed an AGP. Our data are incomplete, but these low levels of influenza contamination on FFRs supports the recommended guidance for the use of FFRs in healthcare settings [8, 38]. It should be noted that although low levels of influenza contamination were detected on FFRs that were used during AGPs, CDC’s current recommendation is to discard FFRs if they were worn during an AGP [38]. Furthermore, we did not use a random sample (participants are not representative of the makeup of the AED), and we had small number of participations by some of our occupational groups. It is likely that we identified some populations such as nurses who were more likely exposed. In addition, the sampler used collects all airborne particles capable of penetrating the respiratory tract (inhalable fraction), including large droplets. Therefore, we are not able to assess if exposure is predominantly to droplets or aerosols. Finally, we could not determine the source of the virus (whether it came from the environment or the participant) and we could not link whether the contamination led to infection in these HCP.

Still this study is important for a number of reasons. One of the main strengths of our study is the large number of samples collected and analyzed and the fact that we had access to an emergency department and personnel throughout the influenza season, which made possible sampling during the peak of the season. Furthermore, samples were collected during normal operation of an emergency department, reflecting real-life exposures, and use of personal bioaerosol samplers on HCP allowed us to get close to the source (patients). Another strength of our study was having access to results of a rapid test program carried out in the adult emergency department that let us know which patients had confirmed influenza, which HCP had interaction with that patient, and which room(s) the patient was seen in within the emergency department. Also, the layout of this emergency department allowed us to sample in an area where patients with influenza like illness are held for day-long observations, increasing our chance to monitor during patient encounter. Finally, we were able to recruit a wide range of occupations, which allowed us to assess the risk of different job titles and job tasks. Participant feedback to the use of the personal samplers was very positive.

Our findings suggest that influenza transmission in healthcare may be multi-modal, as we observed opportunities for exposures via airborne and contact routes. Exposure to influenza can occur throughout the influenza season for a variety of HCP occupations and locations within a healthcare facility. We found certain occupations were at higher risk of exposure and should be targeted for training about appropriate infection prevention activities. FFRs may become contaminated with influenza when used; however, high levels of contamination are unlikely given low aerosol concentrations and short wear times.

## Supporting information

S1 Table Job titles and job descriptions. (DOCX)

S1 Fig Participants’ daily survey. (DOCX)
