# Supplementary material for: Healthcare personnel exposure in an emergency department during influenza season
Source: PLoS One. 2018 Aug 31;13(8):e0203223. doi: 10.1371/journal.pone.0203223 (PMC6118374; doi:10.1371/journal.pone.0203223)
Supplement: S1 Table — (DOCX) [file pone.0203223.s001.docx]

**S1 Table. Job titles and job descriptions**

| Job Title | Job Description |
| --- | --- |
| Support staff  (Unit Associate) | Provides a variety of unit operational support and activities to promote patient comfort and satisfaction.  Works under the direction of a Registered Nurse. |
| Nursing Shift coordinator | The Nursing Shift Coordinator is responsible for facilitating delegated administrative and clinical activities for nursing in a functional unit.  This person possesses the clinical and management knowledge and ability to provide guidance and assistance to nursing personnel and other health care team members in patient care situations.  He/she facilitates interpretation/implementation of policies and standards. |
| Registered Nurse (Nurse Clinician I) | The Nurse Clinician I is responsible for coordinating care for assigned patients on a shift to promote the achievement of clinical outcomes, providing evidence-based patient care based on the nursing process policy, procedures, and protocols of the Johns Hopkins Hospital, working collaboratively with a multidisciplinary health care team to optimize team performance, participating and promoting quality clinical improvement on unit by supporting departmental and hospital safety standards, and practicing responsible cost-effective use of resources. |
| Nursing supervisor (Nurse Manager) | Provides leadership to achieve unit, departmental, and institutional objectives within a rapidly changing environment. Applies advanced knowledge of nursing and management to manage systems of patient care, personnel development, fiscal and unit operations, performance improvement, and program development. Promotes innovation and excellence in clinical, service, and team performance. |
| Clinical technician | Under the direct supervision of a Registered Nurse, and taking into consideration the developmental needs of the patient, performs routine patient activities and delegated patient care procedures of a specialized and technical nature which includes observing and monitoring patient condition, identifying changes in collected patient data and notifying nurse, setting‐up and maintaining specialized clinical equipment, knowledge of aseptic technique, collecting blood specimens and performing related, delegated nursing procedures. |
| Certified nursing assistant | Performs and documents delegated patient care activities under the direct supervision of a Registered Nurse. Performs all patient care activities as defined in the skills competency model with compassion, dignity, and respect. Performs patient care activities with scope of practice and according to JHH clinical standards. Performs unit-based technical activities as outlined in the unit- based skills competency model. Documents and reports data collection activities contributing to patient assessment. |
