# Supplementary material for: Healthcare personnel exposure in an emergency department during influenza season
Source: PLoS One. 2018 Aug 31;13(8):e0203223. doi: 10.1371/journal.pone.0203223 (PMC6118374; doi:10.1371/journal.pone.0203223)
Supplement: S1 Fig — (DOCX) [file pone.0203223.s002.docx]

**S1 Figure. Participants’ Daily Survey**

N95-N95 Filtering Facepiece Respirator

FPE-Facial Protective Equipment
